# Quantifying Syringe Exchange Program Operational Space in the District of Columbia

**DOI:** 10.1007/s10461-016-1405-y

**Published:** 2016-04-19

**Authors:** Sean T. Allen, Monica S. Ruiz, Jeff Jones

**Affiliations:** 1 Department of Prevention & Community Health, Milken Institute School of Public Health, The George Washington University, 950 New Hampshire Ave, Suite 300, Washington, DC 20052 USA; 2 Department of Health Policy & Management, Jiann-Ping Hsu College of Public Health, Georgia Southern University, PO Box 8015, Statesboro, GA 30460 USA; 3 Department of Epidemiology, Johns Hopkins University Bloomberg School of Public Health, 615 N. Wolfe St., Room E6534, Baltimore, MD 21205 USA

**Keywords:** Structural interventions, Syringe exchange programs, Health policy, HIV, Persons who inject drugs

## Abstract

Syringe exchange programs (SEPs) are effective structural interventions for HIV prevention among persons who inject drugs. In 2000, a buffer zone policy (the 1000 Foot Rule) was implemented in Washington, DC, that prohibited SEP operations within 1000 feet of schools. We examined changes in the amount of legal SEP operational space over time. We used data pertaining to school operations and their approximate physical property boundaries to quantify the impact of the 1000 Foot Rule on legal SEP operational space from its implementation in 2000–2013. Adherence to the 1000 Foot Rule reduced SEP operational space by more than 50 % annually since its implementation. These findings demonstrate the significant restrictions on the amount of legal SEP operational space in Washington, DC, that are imposed by the 1000 Foot Rule. Changing this policy could have a significant impact on SEP service delivery among injectors.

## Introduction

In the United States (US), an estimated 2.6 % (approximately 6,612,488 persons) of the population has ever injected drugs [1]. This is a concerning estimate given the disproportionate burden of human immunodeficiency virus (HIV) and hepatitis C virus (HCV) infections among injectors [2]. Since the 1980s, the number of new HIV infections attributed to injection drug use (IDU) has declined, yet new cases are still diagnosed among persons who inject drugs (PWID) both domestically and globally [3]. Globally, HCV prevalence rates among PWID range from 20 to 50 % [4]. In the United States, IDU is the most common route of HCV transmission [5].

According to epidemiological data collected through the end of 2012, an estimated 16,702 persons are living with HIV in the District of Columbia (DC) [6]. In 2007, IDU accounted for 149 new cases of HIV in DC and was the third leading cause of transmission, with the men who have sex with men (MSM) and heterosexual sex exposure categories ranking first and second, respectively [7]. Since 2007, the number of IDU-associated new infections has decreased dramatically, with only 30 cases attributable to IDU in 2011 [6]. While the HIV transmission rates from IDU exposure have declined, HCV transmission continues to be a concern. Between 2008 and 2012, 15,915 new cases of chronic HCV infection were diagnosed [6]. Further, research has found that, during a 2010 survey of DC PWID, 90 % of participants indicated they were HCV positive [8].

The injection drug use epidemic is difficult to address given the complexities of addiction and limitations of existing drug treatment modalities. A 12-year longitudinal study of PWID found that 14.3 % had experienced a single relapse and 36.9 % had experienced multiple relapses [9]. Multiple studies have found that PWID require several attempts in drug treatment programs before they will achieve sustained substance use cessation [10, 11]. Other research has shown that substance users who are retained in treatment programs for a year or longer are nearly five times more likely to have better outcomes regarding reduced illicit drug use, alcohol use, and criminal involvement compared to their counterparts who are in treatment programs for less than a year [12]. Given that a range of factors may influence the willingness and ability of PWID to engage in treatment programs, it is important that public health interventions go beyond drug treatment programs to address health outcomes among this population.

Structural interventions for HIV prevention are one example of effective public health practice for PWID. These interventions refer to policies and programs that change environments in which health risks occur without attempting to change the knowledge, attitudes, or other social interactions of persons at risk [13]. Needle and syringe exchange programs (SEPs) are structural interventions that are of significant importance to injector populations. SEPs have been shown to reduce the incidence of HIV and HCV among injectors via provision of sterile injection equipment and to reduce risky injection behaviors, resulting in significant cost savings in treatment costs from averted HIV infections [14–19]. For example, a 2015 study in Washington, DC, found that the removal of a policy that blocked municipal funding of SEPs—thereby allowing subsequent implementation of a network of syringe access services—resulted in an estimated 120 averted HIV infections in the two years following the policy change. This number of infections averted corresponded to a 45.6 million USD savings in lifetime HIV treatment costs [19]. Beyond the provision of sterile injection equipment, SEPs may provide necessary health care services (such as HIV and HCV testing) onsite as well as referrals to other medical and social services (e.g., screenings for sexually transmitted infections, substance use treatment, etc.). SEPs may also provide health education on topics that are relevant to PWID, such as how to engage in safer injection practices (e.g., not sharing injection equipment, how to sterilize syringes, etc.) and how to prevent overdose [20, 21]. Though engagement with SEPs is not equivalent to unique encounters for health care, SEPs do provide critical health services to a population that may otherwise not receive medical care in traditional healthcare facilities due to stigmatization and/or criminalization of their behavior.

There is an abundance of empirical literature documenting the public health benefits of SEPs [14–19], yet policies pertaining to their implementation may not be grounded in research evidence [22]. Policy level impediments to SEP implementation have a history of complicating harm reduction service provision in the US. For example, in 1914 the Harrison Act was passed which made the possession of injection equipment illegal without a prescription. Many states have passed similar paraphernalia legislation, including polices that govern the sale of sterile injection equipment by pharmacies [21]. Another example of a policy-level impediment to SEP efficacy occurred in 1988 when Congress passed legislation (known as “The Federal Ban”) prohibiting the use of federal monies to support SEPs [23–25].

The implementation of buffer zone policies has also produced significant barriers to SEP operations. Buffer zone policies limit where SEPs can legally operate in a given geographical area. For example, SEP operations were prohibited within 1500 ft. of schools in Pittsburgh, PA; SEP operations were prohibited within 1000 ft. of a school or day care center in Denver, CO [26–29]. Though the buffer zone policies in Pittsburgh and Denver were repealed in 2014 and 2013, respectively, [26–29], they still exist in other cities.

In the District of Columbia, SEP operations are subject to buffer zone restrictions. In 2000, the DC government passed the 1000 Foot Rule (§48-1121), prohibiting the distribution of “any needle or syringe for the hypodermic injection of any illegal drug in any area of the District of Columbia which is within 1000 feet of a public or private elementary or secondary school (including a public charter school)” [30]. This policy may impede SEP accessibility for DC injectors, a population that, on average, may travel approximately 3 miles to access harm reduction services [31] and is often marginalized from traditional sources of health care.

Although it has been in place since 2000, no research has examined the impact of the 1000 Foot Rule on the amount of land in DC that is legally available for SEP operations. Further, no research has quantified the amount of legal SEP operational space in the District via use of the physical property boundaries of schools. This is a significant gap in the literature that warrants exploration given that PWID may serve as a bridging population to other groups through shared paraphernalia use, sexual network interactions, etc. and that the 1000 Foot Rule may effectively prohibit SEP operations in the majority of the District. Restricting the amount of legal space for SEP operations could cause the SEPs to operate in areas that are not in proximity to areas of relevance to PWID populations and, as a result, lead to injectors not having sufficient access to sterile injection equipment. The purpose of this descriptive research was to examine the effect of the 1000 Foot Rule (from its implementation in 2000–2014) on legal SEP operational space (i.e., areas in which SEP operations are not restricted by the 1000 Foot Rule) in DC. We hypothesized that the amount of legal SEP operational space would decline annually as a result of the proliferation of schools.

## Methods

School operations data from 2000 through the 2013 school year were accessed via publicly available sources (e.g., annual reports, school directories, etc.), online searches, and Freedom of Information Act (FOIA) requests to the DC Public Charter School Board and DC Public Schools. Data were also abstracted from publicly available resources and datasets created by the National Center for Education Statistics, the DC Office of the Chief Technology Officer (OCTO), http://www.education.com, and The National Association of Independent Schools [32–35]. Collectively, these data sources allowed for a comprehensive historical accounting of all schools that operated in the District from 2000 to 2013. The school operations data extracted from these sources included: address of the schools, what grade(s) were taught during each school year, and what year(s) the schools were in operation.

School operations data were aggregated into a single dataset in Microsoft Excel. Because the school operations data were derived from a number of sources that did not necessarily limit their data collection to public or private schools in DC, all data were inspected manually to determine if the location met the criteria of the 1000 Foot Rule (i.e., public or private elementary or secondary schools, including public charter schools that operated in DC). All locations that did not meet the criteria of the 1000 Foot Rule were excluded from the analyses, such as facilities that only offered childcare (pre-kindergarten services) or operated in adjacent metropolitan areas in Virginia and Maryland. Academic facilities within detention centers were also excluded from the analyses, as they did not represent traditional conceptualizations of schools where youth have personal autonomy. Lastly, private company “learning centers” were also excluded from the analyses as they did not fit the definition of a school but were, instead, for-profit academic enhancement centers that offered supplemental tutoring/academic counseling.

The DC Master Address Repository (MAR) Geocoder, a publicly accessible tool that allows the user to search a database of addresses, blocks, intersections, place names and other location identifiers in the District, was used to download geographic data about each school [33]. These data included the Square Suffix Lot (SSL) identifier for each location. The SSL identifier is used by the DC Government for city planning processes and taxation assessments. A dataset of approximate land parcel boundaries (including SSL data) was downloaded from the DC GIS Data Clearinghouse [36]. The output from the DC MAR application was then matched to the school property dataset using the SSL identifier. ArcMap v10.2.1 was used to extract the approximate property boundaries of each school. Schools that did not generate a match between the two datasets were geocoded to their approximate physical address location in order to limit the influence of missing property boundary data on the analyses. For non-matched locations, the geocoded location resulted in a point on the map denoting the school rather than the approximate property boundaries.

The academic year was used to frame the analyses because of its direct impact on the application of the 1000 Foot Rule. More specifically, the policy implications are dependent on school operational years (i.e., when school is in session) rather than fiscal or calendar years. All steps in the mapping process were repeated for each academic year of interest. Further, all analyses were completed at the city-level due to shifts in the ward boundaries over the study period.

All areas where SEP operations could not occur due to policy restrictions other than the 1000 Foot Rule (e.g., areas under Federal jurisdiction, such as national parklands and military installations) were quantified using ArcMap by academic year. Bodies of water were also quantified as SEP ineligible areas as they pose obvious geographic impediments to service delivery. The square mileage of each of these areas was calculated at the city level during each academic year. These areas were aggregated into a single continuous layer via the Merge and Dissolve tools in ArcMap. This allowed for the elimination of potential overlap between them (e.g., bodies of water located in national parklands) and a more accurate quantification of the potential SEP operational space (i.e., areas where SEPs could operate in the absence of the 1000 Foot Rule).

After completing the preliminary mapping of potential SEP operational space, ArcMap was used to measure the amount of land space in which SEPs could operate after taking into account the 1000 Foot Rule. A 1000-foot buffer was applied to school property boundaries and to the point location of those schools that did not generate a match to DC MAR data. School buffers were combined into a single continuous layer via the Merge and Dissolve tools in ArcMap. Because some of the buffers extended beyond the boundaries of DC, the Clip tool was used to tailor all analyses to only those areas within the boundaries of DC.

To most accurately reflect the impact of the 1000 Foot Rule on the amount of legal SEP operational space, the clip tool was used to tailor the analyses of the buffer zones such that their quantifications excluded areas where they overlapped with regions that were not eligible for SEP operations due to reasons other than the 1000 Foot Rule (i.e., water bodies, military installations, and national parklands). ArcMap was then used to calculate the total square mileage of the potential SEP operational space that was ineligible for SEP activities due to the 1000 Foot Rule by academic year. The percentage impact of the 1000 Foot Rule on potential SEP operational space was calculated at the city level (i.e., the percent of the potential SEP operational space that was ineligible for SEP operations due to the 1000 Foot Rule). These data were then graphed to show the impact of the 1000 Foot Rule on SEP operational space over time.

## Results

The District of Columbia is approximately 68.5 square miles. Bodies of water, military installations, and national parklands occupy 7.26, 2.80, and 10.73 square miles, respectively. After merging and dissolving these three layers into a single continuous layer to eliminate any potential overlap, their collective area occupied 20.47 square miles (29.88 %) of DC. The exclusion of these areas left 48.05 square miles as potential SEP operational space (i.e., areas where SEPs could legally operate in the absence of the 1000 Foot Rule).

In total, 287 unique properties were identified as school locations that operated in DC during at least one academic year of the study period. Of these, 97.9 % (n = 281) generated matches to the DC MAR Geocoder. After applying a 1000-foot buffer to the school property boundaries (n = 281) and point locations of schools that did not generate matches (n = 6), the total amount of overlap between these areas and the aggregated layer of water bodies, military installations, and national parklands was calculated by academic year. The amount of this overlap ranged from 2.48 to 2.91 square miles. After subtracting the overlap of these areas, the impact of the 1000 Foot Rule on potential SEP operational space remained. The total square mileage of the potential SEP operational space the 1000 Foot Rule caused to be ineligible for SEP activities held approximately constant over the study period, ranging from 24.30 to 25.83 square miles (50.57–53.76 % of the total area of DC).

**Fig. 1:**
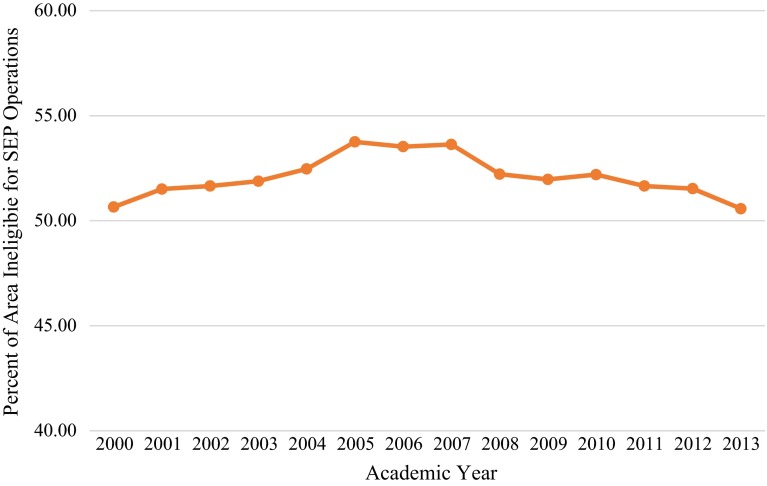
Percent of potential SEP operational space ineligible for SEP operations due to 1000 Foot Rule

**Fig. 2:**
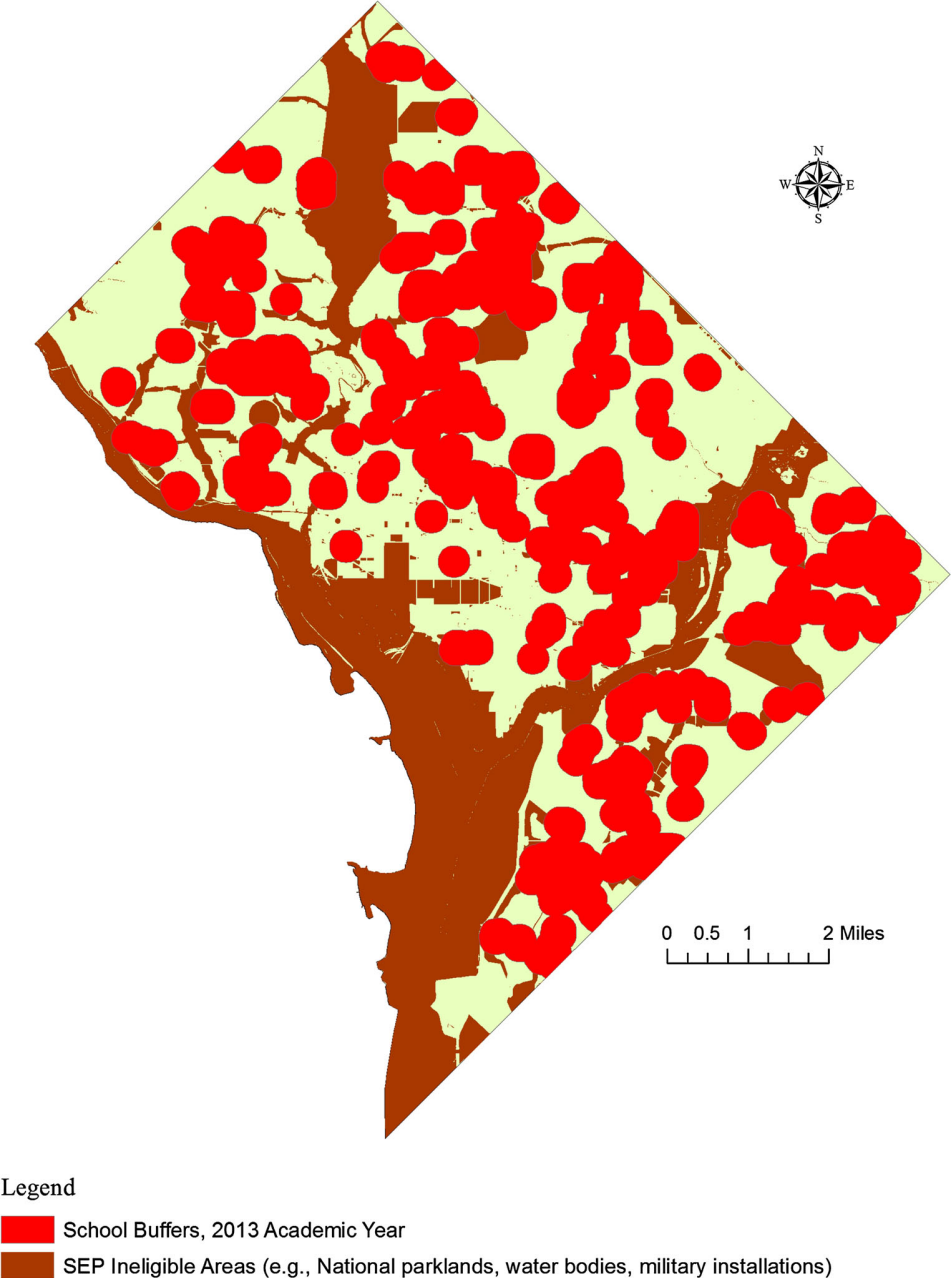
Areas ineligible for SEP operations (2013 academic year) in Washington, DC

Notably, in 2000 (the year of the policy implementation), 50.66 % of the potential SEP operational space was ineligible for SEP operations. Thirteen years later (2013), 50.57 % of the potential SEP operational space was ineligible for SEP operations. These data do not support the hypothesis that the amount of land ineligible for SEP operations would increase over time as a result of more schools opening. Although the number of schools increased over the study period, the overall size of the aggregated school buffers changed very little due to the amount of overlap between the school buffers. This finding is explained by the fact that the school buffers were so expansive at the time of policy implementation that the opening of new schools had a negligible effect on the amount of overall SEP operational space in the District. These data are summarized in Table 1 and Fig. 1. An exemplar image of the effects of the 1000 Foot Rule on legal SEP operational space is depicted in Fig. 2.

**Table 1:** Impact of 1000 Foot Rule on SEP operational space in DC

Academic year	Total number of schools in operation	Number of schools matched to property records	Number of schools geocoded by address alone	Area (square miles) ineligible for SEP operations due to 1000 Foot Rule	Percent of Total Area of Potential SEP operational space ineligible for SEP services due to 1000 Foot Rule
2000	224	218	6	24.34	50.66
2001	227	221	6	24.75	51.51
2002	229	223	6	24.82	51.65
2003	232	226	6	24.93	51.88
2004	243	237	6	25.21	52.47
2005	250	244	6	25.83	53.76
2006	254	249	5	25.72	53.53
2007	256	250	6	25.77	53.63
2008	250	245	5	25.09	52.22
2009	251	246	5	24.97	51.97
2010	252	247	5	25.08	52.20
2011	248	243	5	24.82	51.65
2012	248	243	5	24.76	51.53
2013	250	245	5	24.30	50.57

## Discussion

This descriptive analysis of the effect of the 1000 Foot Rule on legal SEP operational space demonstrates the potential impediments SEP providers must navigate in their service delivery activities. It was hypothesized that the square mileage of land ineligible for SEP operations due to the 1000 Foot Rule would increase over time as a product of more schools opening, but the data did not support this hypothesis. The percentage of the potential SEP operational space that fell within 1000 feet of a school remained near 50 % (range 50.57–53.76 %) over the study period. These data suggest that that when the 1000 Foot Rule was implemented, the schools were already so numerous and geographically dispersed that the addition of more schools had very little impact on the overall amount of available lands eligible for legal SEP services. In other words, the 1000 foot buffer coverage areas were so large that they overlapped with those of the new schools that opened over time. This finding was illustrated by comparing the effect of the 1000 Foot Rule in the 2000 and 2010 years on potential SEP operational space. In these years, there were 224 and 252 schools in operation, respectively, yet the buffers of these locations resulted in the ineligibility of only 50.66 and 52.20 %, respectively, of the overall potential SEP operational space.

Between some academic years, the number of schools increased while the percentage of SEP operational space affected by the 1000 Foot Rule decreased. This fact is explained by the degree of overlap between the buffers of those schools that opened/closed over the years and those that remained in operation. In these scenarios, the net impact of the increasing number of schools was negative due to the amount of overlap among the school buffers and the simultaneous closing of some school locations and associated decreases in the amount of land space ineligible for SEP operations.

It is important to note that the data do not explore the extent to which the 1000 Foot Rule directly affected SEP service provision and the health of DC PWID. The 1000 Foot Rule may exist in legal terms, but have minimal impact on actual SEP service delivery due to limitations in the abilities of SEP providers to comprehensively account for all school properties and their associated buffers zones. In other words, SEPs may attempt to abide by the 1000 Foot Rule, but do so in ways that do not fully account for the application of the 1000-foot buffer to the physical property boundaries of schools. Importantly, the DC government does not provide maps to the SEP service providers illustrating the legal space where they may engage their clients. Given that there are multiple SEP service providers in the District, this policy may lead to uneven service delivery because harm reduction organizations have no guidance on the exact locations that are legal for SEP operations. Additionally, without such clear guidance, each SEP provider may be interpreting the 1000 Foot Rule differently. With an ever-changing landscape of school sites, it is critical that both harm reduction providers and government officials collaborate to develop clear service implementation plans that both optimize SEP service delivery in areas of greatest need and maintain the legality of service provision.

Despite the limitations the 1000 Foot Rule imposes on the amount of lands available for legal SEP operations, the number of new HIV infections attributed to IDU in the District has continued to decline over time. In 2008, there were 109 new HIV infections among PWID; in 2012, there were only 21 new HIV infections among PWID [6, 8]. As noted in other research, decreases in the number of new HIV infections among PWID may be explained by efforts of the DC Department of Health to increase HIV awareness and testing [19]. The decrease in HIV incidence among PWID could also be explained by the proliferation of SEP providers. From 1996 to 2008, a single SEP existed in the District. In May 2008, a network of SEPs was created that dramatically increased the provision of sterile injection equipment among PWID [19]. While the decrease in HIV incidence among PWID is a noteworthy success, more work needs to be done to reach zero new infections.

Reforming the 1000 Foot Rule could enable SEP providers to access portions of the PWID population that are not currently engaged at harm reduction providers due to access barriers. Future work should explore the impact of the 1000 Foot Rule on legal SEP operations in areas of greatest need (e.g., such as wards with high HIV incidence or in areas with disproportionate rates of substance use). Another area of future work should include developing innovative strategies that enable persons who reside in these locations to consistently access sterile injection equipment without compromising the quality or legality of service provision. Lastly, because we cannot ascertain the degree to which the 1000 Foot Rule directly affected the utilization of SEP services among DC PWID, research should be undertaken to qualitatively explore the degree to which the policy affected SEP engagement among injectors and how rigorously SEP providers abided by the policy restrictions.

Another noteworthy consideration for the interpretation of these data pertains to the inclusion of the 2000 academic year data. The 1000 Foot Rule did not go into effect until “120 days after November 22, 2000” [30]. The 2000 academic year was included due to the partial applicability of the 1000 Foot Rule to this time period. Any retrospective applications of study data should take the date the policy went into effect under consideration.

This descriptive research makes a notable contribution to the public health literature in that it quantified the amount of legal SEP operational space after application of the 1000 Foot Rule to the physical property boundaries of schools. No research has documented the changes in the percent of legal SEP operational space over time. The methodology used in this research could be integrated into comprehensive studies of buffer zone policies that evaluate the impact of their implementation and removal on HIV incidence among PWID.

A strength of this research is its utilization of DC government datasets to better understand the actual impact of the 1000 Foot Rule on SEP operational space. This study abstracted the approximate physical property boundaries for use in the analyses. Further, in using these data, nearly 100 % of the school locations generated matches to the property boundary dataset. Schools that did not generate matches (n = 6) were geocoded manually and a 1000 foot buffer was applied to the point location of the physical address of the schools. As such, we are confident this research provides a comprehensive accounting of SEP operational space in the District.

A limitation of this research is that not all schools that were in operation in DC during the study period may have been identified. In addition to the operations data released by DC Public Schools and the DC Public Charter School Board, thorough searches of online sources were conducted to identify schools. However, schools that ceased operations may have a diminished presence on online sources and may not have been identified for inclusion in this research. Despite this limitation, given the breadth of data available, we feel this research provides an accurate quantification of the effect of the 1000 Foot Rule on legal SEP operational space.

## Conclusion

In conclusion, the results of this descriptive analysis demonstrate that the 1000 Foot Rule has reduced the amount of legal space available for SEP operations in DC by more than 50 %. This reduction in operational space has remained mostly constant, despite the opening/closing and proliferation of schools. These data provide a starting point for future studies that more comprehensively explore how buffer zone policies directly affect SEP service delivery and, consequently, HIV incidence among PWID. The removal of this policy restriction on DC SEPs could dramatically change where SEP operations occur and help address unmet needs among injectors.

## References

[1] Lansky A, Finlayson T, Johnson C (2014). Estimating the number of persons who inject drugs in the united states by meta-analysis to calculate national rates of HIV and hepatitis C virus infections. PLoS One.

[2] Centers for disease control and prevention (cdc). Hiv surveillance in injection drug users (through 2011) [power point slides]. www.cdc.gov/hiv/library/slidesets/index.html. accessed 14 Aug 15.

[3] Grigoryan A, Shouse RL, Durant Td (2009). HIV infection among injection–drug users—34 states, 2004–2007. MMWR Morb Mortal Wkly Rep.

[4] Aceijas C, Rhodes T (2007). Global estimates of prevalence of HCV infection among injecting drug users. Int J Drug Policy.

[5] Centers for Disease Control and Prevention (CDC). Hepatitis C FAQs for the Public. http://www.cdc.gov/hepatitis/c/cfaq.htm. Accessed 10 March 15.

[6] District of Columbia Department of Health. Annual Epidemiology and Surveillance Report: Surveillance data through 2012. http://doh.dc.gov/sites/default/files/dc/sites/doh/publication/attachments/2013%20Annual%20Report%20FINAL-2.pdf. Accessed 14 Aug 15.

[7] District of Columbia Department of Health, HIV/AIDS, Hepatitis, STD, and Tuberculosis Administration. Annual Epidemiology and Surveillance Report, 2012. http://doh.dc.gov/node/678522. Accessed 10 March 15.

[8] District of Columbia Department of Health HIV/AIDS Administration. Injection Drug Use: IDUs and HIV Infection in DC. http://doh.dc.gov/sites/default/files/dc/sites/doh/publication/attachments/IDU_Behavior_Study_2010__0.pdf. Accessed 14 Aug 15.

[9] Galai N, Safaeian M, Vlahov D, Bolotin A, Celentano DD (2003). Longitudinal patterns of drug injection behavior in the ALIVE Study cohort 1988–2000: description and determinants. Am J Epidemiol.

[10] Bammer G, Battisson L, Ward J, Wilson S (2000). The impact on retention of expansion of an Australian public methadone program. Drug Alcohol Depend.

[11] Raisch DW, Fye CL, Boardman KD, Sather MR (2002). Opioid dependence treatment, including buprenorphine/naloxone. Ann Pharmacother.

[12] Simpson DD, Joe GW, Rowan-szal GA (1997). Drug abuse treatment retention and process effects on follow-up outcomes. Drug Alcohol Depend.

[13] Des Jarlais DC (2000). Structural interventions to reduce HIV transmission among injecting drug users. AIDS..

[14] Wodak A, Cooney A (2006). Do needle syringe programs reduce HIV infection among injecting drug users: a comprehensive review of the international evidence. Subst Use Misuse.

[15] Hurley SF, Jolley DJ, Kaldor JM (1997). Effectiveness of needle-exchange programmes for prevention of HIV infection. Lancet.

[16] Kerr T, Small W, Buchner C (2010). Syringe sharing and HIV incidence among injection drug users and increased access to sterile syringes. Am J Public Health.

[17] Ksobiech K (2003). A meta-analysis of needle sharing, lending, and borrowing behaviors of needle exchange program attenders. AIDS Educ Prev.

[18] Palmateer N, Kimber J, Hickman M, Hutchinson S, Rhodes T, Goldberg D (2010). Evidence for the effectiveness of sterile injecting equipment provision in preventing hepatitis C and human immunodeficiency virus transmission among injecting drug users: a review of reviews. Addiction..

[19] Ruiz MS, O’Rourke A, Allen ST (2015). Impact evaluation of a policy intervention for HIV prevention in Washington. DC. AIDS Behav..

[20] Avert.org website. Needle and Syringe Programmes (NSPs) for HIV Prevention. http://www.avert.org/needle-and-syringe-programmes-nsps-hiv-prevention.htm. Accessed 14 Aug 15.

[21] Centers for Disease Control and Prevention (CDC). Syringe Exchange Programs [Fact Sheet PDF]. http://www.cdc.gov/idu/facts/aed_idu_syr.pdf. Accessed 14 Aug 15.

[22] Allen ST, Ruiz MS, O’Rourke A (2015). The evidence does not speak for itself: the role of research evidence in shaping policy change for the implementation of publicly funded syringe exchange programs in three us cities. Int J Drug Policy.

[23] Belani HK, Muennig PA (2008). Cost-effectiveness of needle and syringe exchange for the prevention of HIV in New York City. J HIV AIDS Soc Serv..

[24] Holtgrave DR, Pinkerton SD, Jones TS, Lurie P, Vlahov D (1998). Cost and cost effectiveness of increasing access to sterile syringes and needles as an HIV prevention intervention in the United States. J Acquir Immune Defic Syndr Hum Retrovirol..

[25] Green TC, Martin EG, Bowman SE, Mann MR, Beletsky L (2012). Life after the ban: an assessment of US syringe exchange programs’ attitudes about and early experiences with federal funding. Am J Public Health.

[26] Asmar M. Syringe exchange: Denver City Council lifts 1,000-foot school buffer. The Denver Post. http://www.denverpost.com/politics/ci_24253791/denver-council-looks-at-eliminating-school-buffer-syringe. Accessed 7 Aug 15.

[27] Allegheny County Health Department. County of Allegheny, Pennsylvania, Ordinance No. 16782, and Allegheny County Health Department Rules and Regulations, Article XXI Air Pollution Control. http://www.achd.net/regulations/Article%20II.pdf. Accessed 7 Aug 15.

[28] Aupperlee A. Needle-Exchange Ban Lifted in City. Pittsburgh Tribune-Review. http://www.highbeam.com/doc/1P2-36925924.html Accessed 7 Aug 15.

[29] Aupperlee A. Allegheny County Council lifts restrictions on needle exchanges in Pittsburg. *TribLive News*. http://triblive.com/news/allegheny/6378156-74/council-county-park#axzz3Gdha1yMT Accessed 7 Aug 15.

[30] § 48-1121 Distribution of needle or syringe near schools prohibited. District of Columbia Official Code. Division VIII, Title 48, Subtitle III, Chapter 11, Subchapter 2. http://dccode.elaws.us/code?no=48-1121. Accessed 7 Aug 15.

[31] Allen S, Ruiz MS, O’Rourke A (2015). How far will they go? Assessing the travel distance of current and former drug users to access harm reduction services. Harm Reduct J..

[32] National Center for Education Statistics. Search for Public Schools http://nces.ed.gov/ccd/schoolsearch/. Accessed 15 Sep 14.

[33] Office of the Chief Technology Officer. Master Address Repository. http://octo.dc.gov/node/715602/. Accessed 15 Sep 14.

[34] Education.com Website. http://www.education.com/. Accessed 15 Sep 14.

[35] National Association of Independent Schools. NAIS School Search http://www.nais.org/Users/Pages/SchoolSearch.aspx?src=topband. Accessed 15 Sep 14.

[36] DC Geographic Information Systems Program (DC GIS) Data clearinghouse/catalog. District of Columbia Government website. http://dcatlas.dcgis.dc.gov/catalog/. Accessed 9 Apr 14.

